# Medical Opinion and Movement

**Published:** 1909-01-16

**Authors:** 


### Medical Opinion and Movement.

Mr. Butlin has published in the *British Medical Journal* a most instructive paper on cancer of the tongue, which he read before the International Surgical Society in Brussels last September. The first point that strikes one is that his entire experience of the operative treatment of this disease includes 197 cases—a number which, though doubtless larger than any other British surgeon can claim, is not so large as might have been anticipated. Mr. Butlin has out of this number 55 successes, by which he means patients who are either alive and well or have died of intercurrent diseases, more than three years after operation. There are also six more who are alive and well between one and three years, and four who died of intercurrent disease within that interval. These results are very decidedly good, and a very instructive table shows how superior they are in the last 99 cases to the first 98. Mr. Butlin began the routine removal of the contents of the anterior triangle in 1895, and has done this in 70 cases in all. Excluding 11 cases which are not yet admissible as successful, and two others for good cause shown, this series shows 24 successes out of 57 cases—a percentage which there need be no hesitation or grudging in describing as most remarkable. As against this are placed the 12 successes in 41 countable cases wherein the triangle was not cleared out, and undertaken since 1895.

So much for statistics. Mr. Butlin's opinions, based upon this series, are, of course, of the greatest value and importance. He prefers to undertake the lymphatic removal at a separate (later) sitting, though when the glands are palpable he not very infrequently does the operation in a single stage. He is strongly in favour of this part of the operation as a routine, whether the glands can be palpated or not. The dissection should be carried well up to the parotid region, and the lower part of the parotid salivary gland removed: if the primary disease is far back on the tongue, the posterior triangle of the neck may be swept clean as well as the anterior. So far as his records go, Mr. Butlin inclines to the belief that it is not essential to clear the anterior triangle of the opposite side; but with the reservation that he has not much evidence on this question. In complicated cases, such as those in which both sides of the tongue or of the neck, or the tongue on one side and the neck on the other are involved he would clean both sides. To Mr. Cheatle's suggestion of removing always the extrinsic muscles of the tongue up to their bony attachments he affords support based upon theoretical considerations rather than on the after results of his own cases. The removal of tongue and glands with all the intervening tissue in one mass he regards as too dangerous in proportion to the advantage gained. Early diagnosis and small growths do not justify the surgeon in performing a less complete operation than in less favourable conditions. It is worthy of note that two patients out of eight operated upon for recurrence in the glands of the neck were cured—a fact which should be borne in mind when tempted to offer the usual gloomy prognosis of this condition. The same issue contains another most interesting communication upon the same subject by Mr. C. P. Childe, whose conclusions are in certain minor details different from those of Mr. Butlin. He considers more especially the technique of the operation: the two papers should certainly be read together. The opinions of these English authorities on this important subject may also usefully be compared with those of Dr. J. C. Warren, as summarised in our issue of November 21, 1908, p. 157.

The earliest known human embryo has until lately been the classical 15-day ovum described originally by Professor Peters, and commonly referred to by his name. This ovum has now yielded pride of place to one which is estimated to be at least a day younger, and is the subject of a memoir by Dr. J. H. Teacher in the *Glasgow Medical Journal*. This observer's opinion about the age of his ovum has been accepted by Peters, after a demonstration of the specimen before the German Anatomical Society in Berlin last year. The problem of reckoning the exact age of this embryo, which is but .77 millimetre in length, is rendered fairly easy by the fact that only one coitus had to be considered, and by the relation in time of this to the expected onset of menstruation. It is established beyond reasonable doubt that the maximum possible age for the Teacher-Bryce embryo is 14½ days, and the minimum 12 days. Dr. Teacher arrives at certain conclusions by a comparison of this embryo with several other very early ones. He agrees with v. Spee that seven days elapse between fertilisation and implantation of the ovum in the endometrium. Fertilisation may occur at any time during the intermenstrual interval, and embedding may take place either during endometrial quiescence or during what would be the premenstrual period but for the occurrence of pregnancy. The menstrual decidua is not a preparation for the ovum, he thinks; nor is menstruation the abortion of an unfertilised ovum. Menstruation is a cyclical process which provides for the maintenance of the endometrium in a suitable condition for producing the decidua of pregnancy, but the ovum is not dependent on a menstrual decidua for a suitable nidus in which to become embedded. He agrees with other authorities that ovulation does not necessarily coincide with menstruation.

The attempt to reduce an intussusception by means of liquids or gases injected per rectum has been condemned by almost all the authorities who have written on this subject during the last few years; and laparotomy as soon as possible after diagnosis is advised as the proper line of treatment. Mr. Aslett Baldwin, however, publishes in the *West London Medical Journal* an account of seven cases treated by irrigation with warm saline solution. He tried this under chloroform anæsthesia, taking care to allow a head of but twelve to eighteen inches of water, and not to add any fluid to the irrigator: by this means he insured a pressure which was continuously becoming less (by the water level in the irrigator sinking) as the bowel became more and more distended and the most damaged part of the bowel was reached. In the first six cases a small exploratory incision was made into the abdomen to ascertain the result of the rectal treatment. It was found that in five cases complete success had been attained: the other case was one in which the symptoms had been present for four days. In the seventh case this precautionary laparotomy was omitted, and the patient did very well. Mr. Baldwin is of opinion that such an operation, which can be done by muscle splitting through a very small incision, is probably advisable in most cases. It is evident, as he says, that shock must be minimised by this irrigation treatment, and on that account alone the plan is worthy of extended trial.

---

Child murder by thrusting a bonnet-pin through the unclosed fontanelle into the brain is the subject of a paper by Bihler in the *Zeitschr. f. Med. Beamte*. This observer had to deal with a girl of fourteen, afterwards pronounced insane, who had, without being detected, thus murdered six infants committed to her charge within a year and a half. She ultimately confessed, but on exhuming the bodies evidence of the injuries she described could be found only in the most recent victim, murdered a fortnight previously. In the others putrefactive changes rendered the injuries invisible. It is pointed out that this form of murder is hardly mentioned in the medico-legal text-books, but that a knowledge of it is fairly widely spread among the laity. Also that the external signs of injury are so slight and the symptoms caused so vague that suspicions of violence are hardly likely to be aroused in those who are called in to treat these cases: certificates of natural death had been issued in all the six cases under review. In the most recently deceased child of the series the evidence gained by post-mortem examination was practically confined to the presence of two small holes in the dura mater and a slight hæmatoma underlying a tiny scab on the skin. The brain was too diffluent for examination.

---

Dr. F. J. W. Maguire, of Detroit, has made a special study of the treatment of typhoid fever in his experience at the United States Marine Hospital, and has recently reported upon his methods in the treatment of 138 consecutive cases of the disease. He is convinced that both in the summer diarrhœa of children and in typhoid fever the administration of nitrogenous food will always cause an immediate rise of temperature. He therefore condemns the usual routine milk diet, and advocates carbohydrates exclusively, in the form of barley or rice water. In eighteen cases he found the temperature rise after a milk feed, while there was no perceptible increase of temperature after rice or barley water. For the first five to ten days he gives only plenty of sterile water until the patient seems to require nourishment, and then he uses well diluted peptonoids and gelatins. His therapeutic treatment consists in rectal injections of weak carbolic acid solutions. He mixes three to five drops of carbolic acid to a pint of sterilised water, and this is allowed to drop into the rectum continuously. The solution is placed in a fountain syringe alongside the bed about a foot above the patient, and the flow is regulated by a gauge with a water glass attachment. He claims that the method is also efficacious in other conditions, such as appendicitis and pneumonia, in reducing the temperature and causing an amelioration of the condition. All the 138 cases of typhoid responded to the treatment successfully. He never had recourse to cold baths. Profuse hæmorrhage occurrred in four cases, but eventually subsided.

---

In recent years several attempts have been made to treat affections of the skin, especially nævi, by causing coagulation in the tissue through exposure to a low temperature by freezing with ether or ethyl chloride. W. B. Trimble has had recourse to liquid air, the evaporation of which causes a more intense coagulation than is obtained by the volatile liquids hitherto used. Professor Pusey, of Chicago, has conceived the idea of employing solidified carbonic acid. This is easily obtained in the form of snow by the evaporation of the liquid carbonic acid. In order to obtain a sufficient quantity of snow for an application, a cylinder of liquid carbonic acid is placed with the cock downwards, around which is attached a pouch of kid. On opening the cock the snow deposits itself in the pouch, from which it can be transferred to the patient, after being pressed and shaped into a piece of the size required. The duration of the application is between ten and thirty seconds. If a rapid destruction of the morbid condition is required and a cicatrix is of little importance, the application may be more prolonged. The process does not generally occasion pain and does not necessitate any anæsthesia.

---

Professor Pusey has treated in this way a large number of nævi, and has always obtained excellent results. A single application of ten seconds is often sufficient to effect a cure. Generally as a result of the application a small scab forms, which falls off at the end of about a fortnight without leaving any scar. Professor Hoffmann, of Berlin, and Dr. Strauss, of Barmen, testify also to having obtained excellent results by this method. Professor Ochsner, of Chicago, has employed the same method for the treatment of angiomata. These tumours require several applications every eight or ten days till the growth has completely disappeared. As soon as the carbonic acid snow is applied a strong constriction of the blood-vessels is seen with complete ischæmia of the tumour surrounded by a zone of hyperæmia. The snow can be applied two or three times on different parts of the angioma at the same sitting. Dr. Sauerbruch, of Marburg, following the same technique, reports some very successful cases of angiomata of the nose, forehead, cheek, and lip, which have been completely cured after a few applications of the carbonic acid snow. He has also used the same method for superficial epitheliomata of the face, and states that the results are in no way inferior to those obtained by the Röntgen rays. On the other hand, the method is simpler, more expeditious, and does not expose the patient to possible complications. In such cases Dr. Sauerbruch finds it necessary to exercise a certain amount of pressure at the centre of the growth, which naturally causes sloughing. At the margin of the growth, however, it is sufficient to apply the snow for a few seconds.

---

In a paper read before one of the French medical societies Dr. Diamantberger seeks to establish a pathological connection between thyroid insufficiency and the various forms of articular rheumatism. The theory is, of course, no new one, and has received some notable adherents, among them Lancereaux, de Quincke, Crozac, and others. Dr. Vincent, of Val-de-Grâce, has described what he calls the "thyroid sign," or thyroid reaction, which he has observed in the course of different infectious diseases, typhoid fever, diphtheria, pneumonia, and acute rheumatism. The reaction consists in a more or less pronounced hypertrophy of one or both lobes of the thyroid gland, and pain and tenderness on pressure of the gland. After the attack the gland may remain hypertrophied, and give rise to symptoms of hyperthyroidism, even Basedow's disease; or in consequence of a sort of functional exhaustion of the gland it may atrophy. In the latter case there arise symptoms of thyroid insufficiency, among which are to be numbered chronic articular rheumatism. The fact that thyroid treatment has given marked relief in some cases of chronic articular rheumatism strongly confirms the theory of thyroid insufficiency; but it is well known now that disappointment is rather the rule, and that the majority of cases do not fall into line with this pathological hypothesis. In the course of the discussion which followed the paper, Dr. Gottschalk took up the uric acid hypothesis and showed that many of the symptoms ascribed to thyroid insufficiency were also claimed by the uric acid pathologists and were shown to accompany the uric acid diathesis. The tempting suggestion naturally follows that the proper solution and elimination of uric acid from the system may depend upon the activity of the thyroid gland and the normal secretion and diffusion of a uric acid solvent, such as thyminic acid.

---

Ureterotomy for calculus has for some time been recognised as an operation of established reputation, and is being rapidly more and more practised. The improvements in the diagnosis of calculus impacted in the ureter, chiefly the advances in skiagraphy, have contributed greatly to the popularity of this operation. A very curious case of simultaneous secondary hæmorrhage from both external iliac arteries following bilateral ureterolithotomy is reported by Moschcowitz in the *Annals of Surgery*, and is worth the attention of those who are interested in this operation. The patient had calculi in the pelvic portions of both ureters, and these were extracted through lateral extra-peritoneal incisions; the ureters were closed with interrupted iodine catgut sutures, and no bleeding occurred at the time of operation. The incisions were drained with slender rubber tubes containing iodoform gauze wicks, and it was the removal of these one week after operation which set up profuse and alarming hæmorrhage. The patient was etherised, and it was then discovered that each drainage tube had ulcerated into the external iliac artery, which was tied above and below the hole on each side of the body. The first hæmorrhage is said to have half filled a two-quart basin, and the second to have been similar. Despite this terrific loss of blood the patient's life was saved, though he was, of course, in a most desperate condition for some time. Pulsation returned in the dorsalis pedis within three days, and in the femoral arteries even sooner. The conclusion which the author of the article draws is that in this locality drainage tubes should be introduced with great circumspection, or better not at all.

---

Professor Hagner is a strong advocate of the operative treatment of gonorrhœal epididymitis, a procedure which seems to have been first practised by Dr. Bazet, of San Francisco. Dr. Hagner's method is as follows: An incision is made through the skin and the parietal layer of the tunica vaginalis at a point over the junction of the epididymis and testicle. The fluid contained in the tunica is thus evacuated, and the enlarged epididymis is examined through the wound, and multiple punctures are made through its fibrous covering with a tenotome, especially where it is enlarged or thickened. If pus escapes through any of these punctures the opening is enlarged with a probe and the cavity washed out with perchloride of mercury solution, succeeded by saline fluid. The testis has meanwhile been delivered through the wound and wrapped in hot cloths. After replacing it, the tunica vaginalis is thoroughly washed out and lightly sewn up with a running catgut suture. Relief of pain and resolution of inflammation are obtained whether pus is found or not, and are ascribed in the latter event to relief of tension and drainage of the distended tunica vaginalis. The after results are remarkable. The operation is only done in the severest cases, but every patient except one has been walking about quite free from pain within a week of its performance. In fact there is instantaneous relief of pain, which has already disappeared when the sufferer recovers from the anæsthetic. Fever, induration, leucocytosis, and other results of acute gonorrhœal inflammation of this region disappear in as remarkable a manner as does pain, and no complications have been noticed. Pus was found 17 times in 21 cases, and of this number the globus minor was affected in 15. Whether the operation diminishes the liability to sterility of the affected organ the operator cannot definitely say, and there is very considerable difficulty in determining the point; but if what is claimed on behalf of it is confirmed by more extended investigation, it is to be imagined that it must have some beneficial action in this respect.

